# Highly Enhanced TMR Ratio and Δ for Double MgO-based p-MTJ Spin-Valves with Top Co_2_Fe_6_B_2_ Free Layer by Nanoscale-thick Iron Diffusion-barrier

**DOI:** 10.1038/s41598-017-10967-x

**Published:** 2017-09-19

**Authors:** Seung-Eun Lee, Jong-Ung Baek, Jea-Gun Park

**Affiliations:** 1 MRAM Center, Department of Electronics and Computer Engineering, Hanyang University, Seoul, 04763 Republic of Korea; 2 MRAM Center Department of Nanoscale Semiconductor Engineering, Hanyang University, Seoul, 04763 Republic of Korea

## Abstract

For double MgO-based p-MTJ spin-valves with a top Co_2_Fe_6_B_2_ free layer *ex-situ* annealed at 400 °C, the insertion of a nanoscale-thickness Fe diffusion barrier between the tungsten (W) capping layer and MgO capping layer improved the face-centered-cubic (f.c.c.) crystallinity of both the MgO capping layer and tunneling barrier by dramatically reducing diffusion of W atoms from the W capping layer into the MgO capping layer and tunneling barrier, thereby enhancing the TMR ratio and thermal stability (Δ). In particular, the TMR ratio was extremely sensitive to the thickness of the Fe barrier; it peaked (154%) at about 0.3 nm (the thickness of only two atomic Fe layers). The effect of the diffusion barrier originated from interface strain.

## Introduction

**Figure 1:**
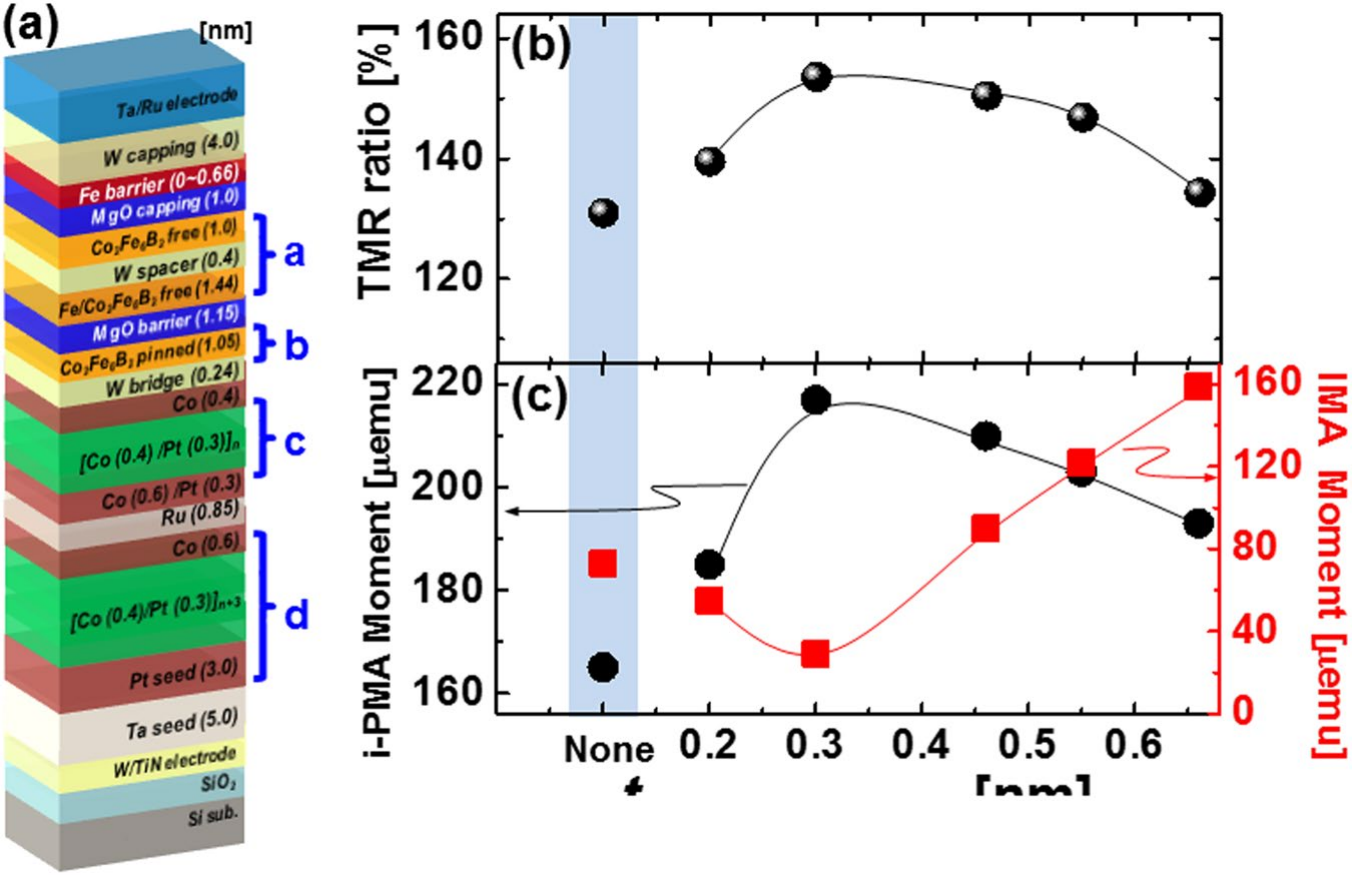
Dependence of TMR ratio on thickness of Fe diffusion barrier for double MgO-based p-MTJ spin-valves with top Co_2_Fe_6_B_2_ free layer. (**a**) p-MTJ spin-valve structure, (**b**) TMR ratio depending on thickness of Fe diffusion barrier, and (**c**) i-PMA and IMA magnetic moment depending on thickness of Fe diffusion barrier.

Perpendicular spin-transfer-torque magnetic-random-access-memory (p-STT MRAM) is a promising memory cell for overcoming the physical scaling limit (~10 nm) of current dynamic-random-access-memory (DRAM) because of its fast write time (~10 ns), non-volatile memory operation, and low power consumption^1–9^. A p-STT MRAM cell consists of a selective device and a perpendicular-magnetic-tunnel-junction (p-MTJ) spin-valve. CoFeB/MgO based p-MTJ spin-valves have been widely researched^10–13^. In particular, for tera-bit-level integration, they require a high tunneling-magneto-resistance (**TMR**) ratio (>150%) for a sufficient write/erase margin, high thermal stability (**Δ** > 74) for a 10-year data retention-time, and a low switching current density (***J***_**c**_ ~1 × 10^2^ MA/cm^2^) for low power consumption at the back end of line (**BEOL**) temperature of 400 °C for such cells^14,15^. Recently, a double MgO-based p-MTJ spin-valve structure with a top CoFeB free layer rather than a bottom CoFeB free layer was proposed as a way of simultaneously achieving a high TMR ratio and high thermal stability at 400 °C (BEOL)^14–17^. So far, structures such as these have used nanoscale-thickness tantalum (Ta) with body-centered-cubic (b.c.c.) crystal structure as the capping layer, spacer layer, and bridging layer. However, it has been extremely difficult to achieve a TMR ratio of >100% at 400 °C (BEOL) because Ta diffusion into the MgO tunneling barrier degrades the f.c.c. crystallinity^18–22^. As a novel solution, the use of nanoscale-thickness tungsten (W) with a b.c.c. crystal structure as a capping layer, spacer layer, and bridging layer, has been proposed a way of enhancing the TMR ratio and thermal stability in double MgO-based p-MTJ spin-valve with a top Co_2_Fe_6_B_2_ free layer; it was found that it could increase the TMR ratio to 134% at an *ex-situ* annealing temperature of 400 °C under a perpendicular magnetic field of 3 tesla (see Supplement 1). Although the TMR ratio was high, it was not quite sufficient for tera-bit-level integration of p-STT MRAM cells^14^. Thus, in this study, we designed a novel double MgO-based p-MTJ spin-valve with a top CoFeB free layer (D-p-MTJ) using W as a nanoscale-thick capping layer, spacer layer, and bridging layer by inserting a nanoscale-thickness b.c.c. crystallized Fe-diffusion barrier between the W capping layer and MgO capping layer, as shown in Fig. 1a.

First, we investigated the effect of the Fe diffusion barrier on the TMR ratio in D-p-MTJs *ex-situ* annealed at 400 °C. The mechanism by which the barrier enhances the TMR ratio was examined by analyzing the static perpendicular-magnetic-anisotropy (PMA) magnetization behavior by using current-in-plane tunneling (CIPT) at room temperature^23^. Then, the f.c.c. crystallinity of the MgO tunneling barrier was observed by high-resolution transmission-electron-microscopy (x-HR-TEM) at 200 keV. The dead-layer thickness and thermal stability were calculated by estimating the PMA magnetic moment using vibrating sample magnetometer (VSM). Lastly, the atomic compositional depth profile of the spin-valve was characterized by using high-resolution secondary-ion-mass-spectroscopy (HR-SIMS: sputtering rate of 0.5 Å/sec), and the strain at the interface between the W capping and MgO tunneling barrier layer through a theoretical calculation.

## Results

### TMR ratios of p-MTJ spin-valves

The dependence of the TMR ratio on the thickness of the nanoscale b.c.c. Fe diffusion barrier was estimated by using CIPT at room temperature in unpatterned films by scanning the perpendicular magnetic field between +300 Oe and −300 Oe^23^, as shown in Fig. 1b and Supplement 2. The TMR ratio was strongly dependent on the diffusion barrier thickness; i.e., it rapidly increased from 131% to 154% as the thickness increased from 0 to 0.30 nm, and it decreased from 154% to 134% as the thickness increased from 0.30 nm to 0.66 nm. Thus, the TMR ratio peaked at a specific diffusion-barrier thickness, i.e., 154% at 0.30 nm. Moreover, the circular-shaped p-MTJ spin-valve cell with a 250-nm diameter had a TMR ratio of 155% and RA of about 10 Ω·*μ*m^2^ at a reading voltage of 0.25 V, which were similar values to those measured by CIPT; see Supplement 3.

### Magnetic properties of p-MTJ spin-valves

**Figure 2:**
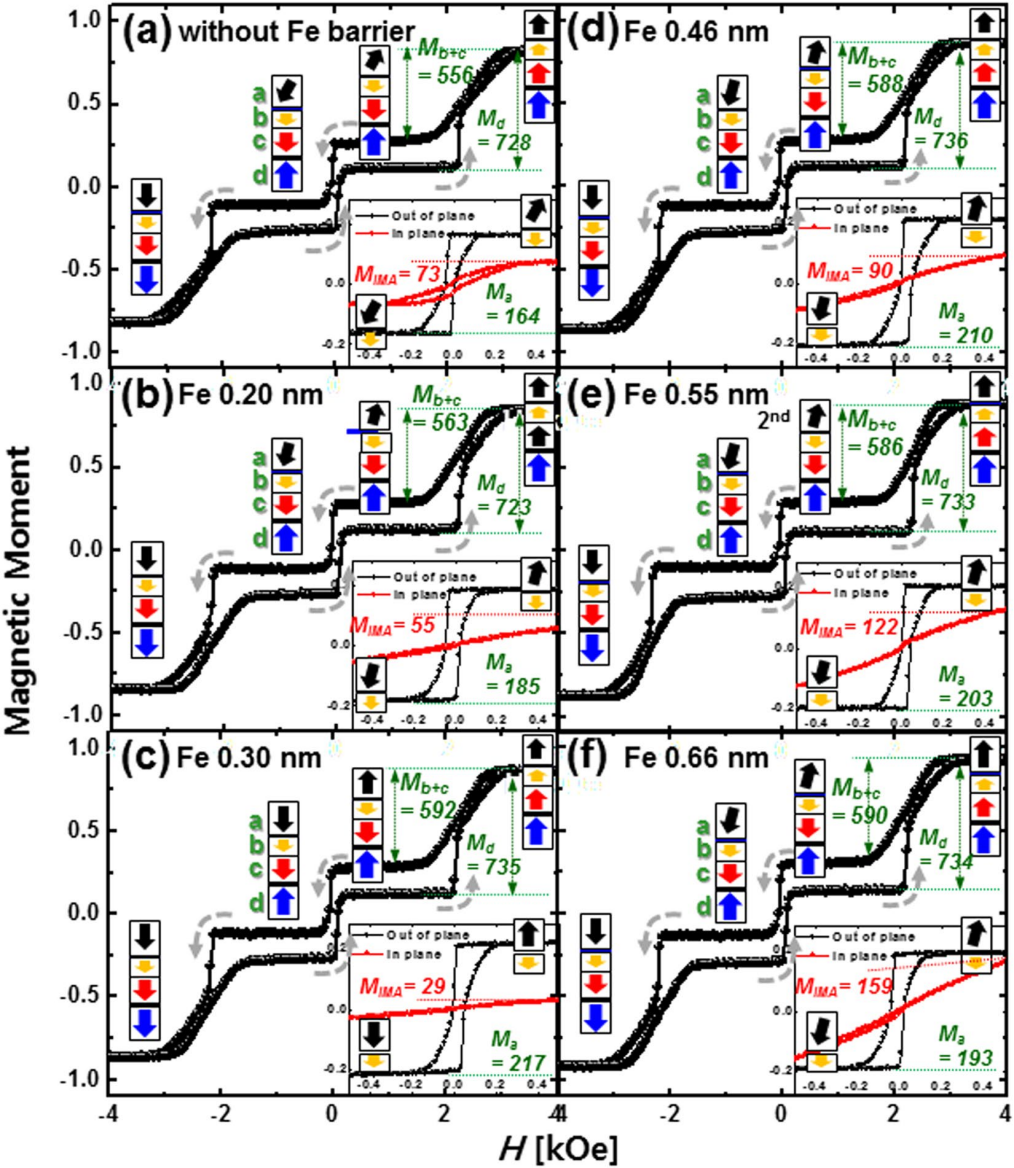
Dependence of static PMA magnetic behavior on thickness of Fe diffusion barrier for double MgO-based p-MTJ spin-valves with a top Co_2_Fe_6_B_2_ free layer. *M-H* curves for p-MTJ spin-valves (**a**) without Fe diffusion barrier, (**b**) with 0.20-nm-thick, (**c**) 0.30-nm-thick, (**d**) 0.45-nm-thick, (**e**) 0.55-nm-thick, and (**f**) 0.66-nm-thick Fe diffusion -barrier. Insets of Fig. 2a–f are *M-H* curves of the Co_2_Fe_6_B_2_ free layer ferro-coupled with the upper Co_2_Fe_6_B_2_ free layer.

**Figure 3:**
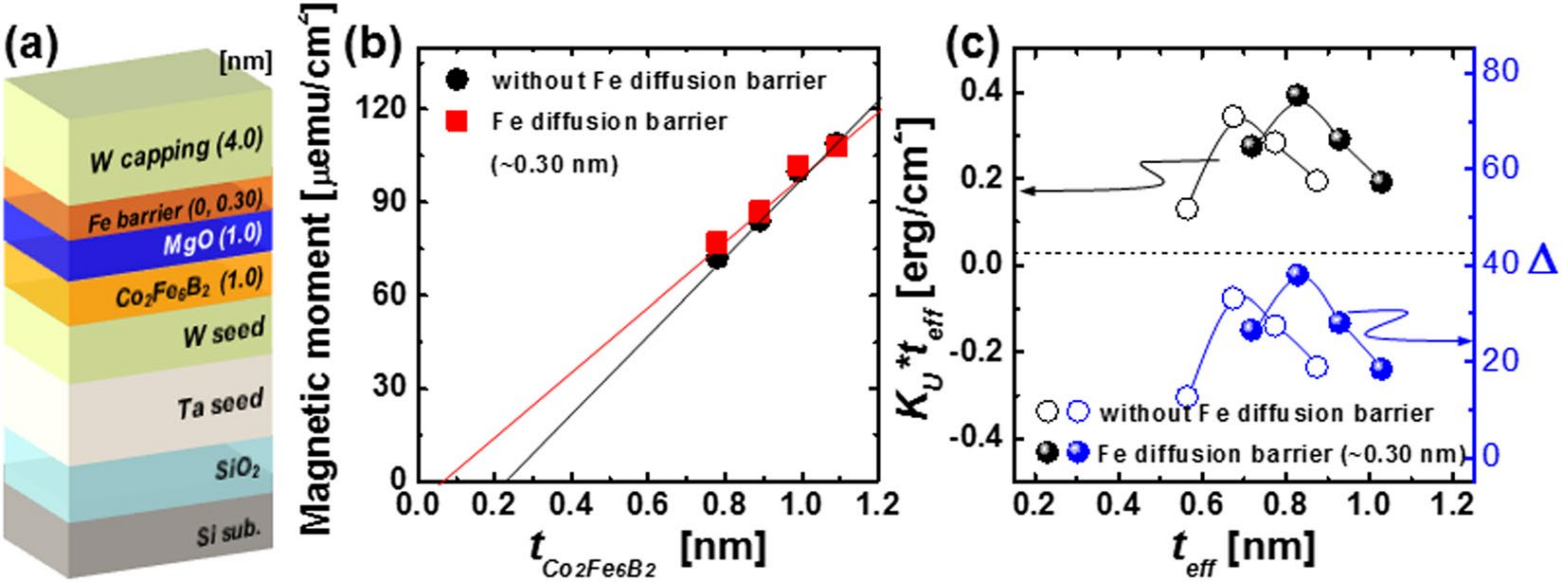
Dependence of thermal stability on thickness of Fe diffusion barrier for PMA structures. (**a**) PMA structure, (**b**) magnetic moment depending on thickness of Fe diffusion barrier, and (**c**) anisotropy energy density and thermal stability depending on thickness of Fe diffusion barrier.

To find out why the TMR ratio peaked at a specific Fe diffusion barrier thickness, we investigated the static magnetic behavior by plotting the magnetic moment versus magnetic field (***M*** **-** ***H*** **)** curves of the spin-valves depending on the barrier thickness, using vibrating sample magnetometer (VSM) (Fig. 2). The magnetic layers of the D-p-MTJs can be largely defined by four regions; double free Co_2_Fe_6_B_2_ layers ferro-coupled via the W spacer layer **a**, Co_2_Fe_6_B_2_ pinned layer **b**, upper SyAF layer **c**, and lower SyAF layer **d**. The Co_2_Fe_6_B_2_ pinned layer **b** was ferro-coupled with the upper SyAF layer **c** via the W bridge layer, while the upper SyAF layer **c** was anti-ferro-coupled with the lower SyAF layer **d** via the nanoscale-thickness Ru spacer layer, as shown in Figs 1a and 2a–f. The lengths of the boxes and vectors in Fig. 2 represent the relative magnitudes of the perpendicular magnetic moment and the spin-electron direction in the magnetic layer^24^. Thus, the *M-H* curves in Fig. 2a–f correspond to the static magnetization behavior of the D-p-MTJ when the applied magnetic field is scanned through 4 kOeto −4 kOe and vice versa. The *M-H* curves in the insets of Fig. 2a–f are the static magnetization behavior of only the Co_2_Fe_6_B_2_ free layers as the applied magnetic field is scanned from + 500 Oe to −500 Oe, which is correlated to the CIPT measurement range. The PMA characteristic of the [Co/Pt]n SyAF layer and interface-PMA (i-PMA) characteristic of the Co_2_Fe_6_B_2_ pinned layer were almost independent of the Fe diffusion barrier; i.e., **M**_**d**_, twice the magnetic moments of the lower SyAF layer, were 728 to 736 μemu, and **M**_**b+c**_, twice the sums of the magnetic moments of the Co_2_Fe_6_B_2_ pinned layer and upper SyAF layer, were 556 to 590 μemu. Note that the p-MTJ spin-valve was designed such that the perpendicular magnetic moment of the lower SyAF layer (**M**_**d**_) was slightly larger than that of the pinned layer ferro-coupled to the upper SyAF layer (**M**_**b+c**_). A thorough examination of the magnetic switching mechanism of the p-MTJ spin valve is presented in our previous paper^24^. However, the i-PMA or in-plane magnetic anisotropy (IMA) characteristic of the upper free layer ferro-coupled with the lower free layer via the W spacer layer considerably depended on the Fe barrier thickness, as shown in the insets of Fig. 2a–f. The i-PMA of the Co_2_Fe_6_B_2_ magnetic layer originated from hybridization of Fe_3d_-O_2p_ and Co_3d_-O_2p_ at the Co_2_Fe_6_B_2_-MgO interface^25^, while IMA is from the bulk of the ferro-magnetic Co_2_Fe_6_B_2_ layer^26^. The i-PMA magnetic moment of the ferro-coupled free layers (**M**_**a**_) rapidly increased from 165 μemu to 217 μemu when the diffusion barrier thickness was increased from 0 to 0.30 nm; it slightly decreased from 217 to 193 μemu when the thickness was further increased from 0.30 nm to 0.66 nm, as shown in Figs 1c and 2a–f, which is explained later. On the other hand, the IMA magnetic moment of the free layers (**M**_**IMA**_) slightly decreased from 73 μemu to 29 μemu when the diffusion-barrier thickness was increased from 0 to 0.30 nm; it rapidly increased from 29 μemu to 159 μemu when the Fe barrier thickness was increased from 0.30 nm to 0.66 nm. Thus, the dependence of **M**_**a**_ on the thickness of the Fe diffusion barrier was inversely related to that of **M**_**IMA**_; i.e., a higher **M**_**a**_ corresponded to a lower **M**_**IMA**_, as shown in Fig. 1c. In addition, the dependence of the TMR ratio on the barrier thickness was well correlated with that of both **M**_**a**_ and **M**_**IMA**_; i.e., a higher **M**_**a**_ and lower **M**_**IMA**_ together corresponded to a higher TMR ratio, as shown in Fig. 1b,c. Although, the dependence of the TMR ratio on the barrier thickness was well correlated with that of both **M**_**a**_ and **M**_**IMA**_, further study on the dependence of the Fe diffusion barrier thickness on the f.c.c. crystallinity for both the MgO tunneling barrier and the MgO capping layer is needed since the TMR ratio is mainly determined by the f.c.c. crystallinity of the two MgO layers. Note that the spin polarization of the perpendicular-magnetic double-free-layers in the p-MTJ spin-valve is dominated by the i-PMA characteristic at the interfaces between the two Co_2_Fe_6_B_2_ free layers and MgO layers, which is directly influenced by the crystallinity of the two MgO layers. The dependence of **M**_**a**_ on the thickness of the Fe diffusion barrier, in which **M**_**a**_ (~217 μemu) peaked at a specific thickness (~0.30 nm), enables us to determine the dead layer thickness and thermal stability of the upper Co_2_Fe_6_B_2_ layer in the p-MTJ spin-valves in Fig. 3. Two kinds of PMA structure, i.e., one without a diffusion barrier and one with a 0.30-nm-thick Fe diffusion barrier, were prepared in order to estimate the dead layer thickness and thermal stability. The Co_2_Fe_6_B_2_ free layer was varied from 0.8 to 1.1 nm and all of the PMA structures were subjected to *ex-situ* annealing at 400 °C for 30 min under a perpendicular magnetic field of 3 tesla (Fig. 3a). For the PMA structure without the diffusion barrier, the dead layer thickness of the Co_2_Fe_6_B_2_ layer was about 0.22 nm, which was consumed by W atoms diffusing from the W capping layer during annealing, as shown in Fig. 3b. For the PMA structure with the diffusion barrier, the dead layer thickness of the upper Co_2_Fe_6_B_2_ layer was approximately 0.06 nm, which means that it was not consumed by W atoms diffusing from the capping layer during annealing. Hence, the barrier worked against the W atoms in *ex-situ* annealing at 400 °C. The anisotropy energy density (*K*_u_ *t*_eff_) was estimated using the dead layer thickness of the upper Co_2_Fe_6_B_2_ layer. The effective thickness is the actual thickness of the Co_2_Fe_6_B_2_ free layer that is responsible for the magnetic properties of the free layer^27^. The anisotropy energy density (~0.34 erg/cm^2^) peaked at an effective free layer thickness of ~0.67 nm for the PMA structure without the diffusion barrier, whereas it (~0.39 erg/cm^2^) peaked at an effective thickness of about 0.83 nm for the PMA structure with the 0.30-nm-thick Fe diffusion barrier (Fig. 3c). These results indicate that insertion of a 0.30-nm-thick Fe diffusion barrier between the W and MgO capping layer enhanced the anisotropy energy density at a greater effective thickness of the upper Co_2_Fe_6_B_2_ free layer. Furthermore, the thermal stability (**Δ**) was calculated by using equation (1).

1 $${\rm{\Delta }}=\frac{{{\boldsymbol{K}}}_{{\boldsymbol{u}}}{\boldsymbol{V}}}{{{\boldsymbol{k}}}_{{\boldsymbol{B}}}{\boldsymbol{T}}}=\frac{{{\boldsymbol{M}}}_{{\boldsymbol{s}}}{{\boldsymbol{H}}}_{{\boldsymbol{k}}}{\boldsymbol{\pi }}{{\boldsymbol{r}}}^{2}{\boldsymbol{t}}}{2{{\boldsymbol{k}}}_{{\boldsymbol{B}}}{\boldsymbol{T}}}$$

Here, *Ms*, *H*_*k*_, *r*, *t*_*eff*_, *k*_*B*_, and *T* are the saturation magnetization, magnetic anisotropy field, radius of the p-MTJ cell, effective thickness of the upper Co_2_Fe_6_B_2_ layer, Boltzmann constant, and temperature, respectively. **Δ** (33.2) peaked at an effective thickness of 0.67 nm for the structure without the diffusion barrier, whereas it (38.0) peaked at an effective thickness of 0.83 nm for the structure with 0.30-nm-thick diffusion barrier, as shown in Fig. 3c and Supplement 4. These results indicate that insertion of a 0.30-nm-thick Fe diffusion barrier between the W and MgO capping layer increased the **Δ** of the upper free layer by approximately 20%, since the barrier prevented diffusion of W atoms during *ex-situ* annealing at 400 °C. Thus, a D-p-MTJ made by inserting a 0.30-nm-thick b.c.c. crystallized Fe diffusion barrier between the W capping layer and the MgO capping layer could achieve enough thermal stability (**Δ** > 74) for a 10-year data retention time at 400 °C (BEOL). Note that the thermal stability in this manuscript was calculated using the effective K_u_ from the unpatterned film. However, the effective Ku usually increases with the decrease of the p-MTJ size down to tens of nm, due to the change of demagnetization energy. Therefore, the Δ for the p-MTJ with nanoscale cell size would be larger than those in our manuscript^28^.

### Crystallinity of the MgO tunnel barrier

**Figure 4:**
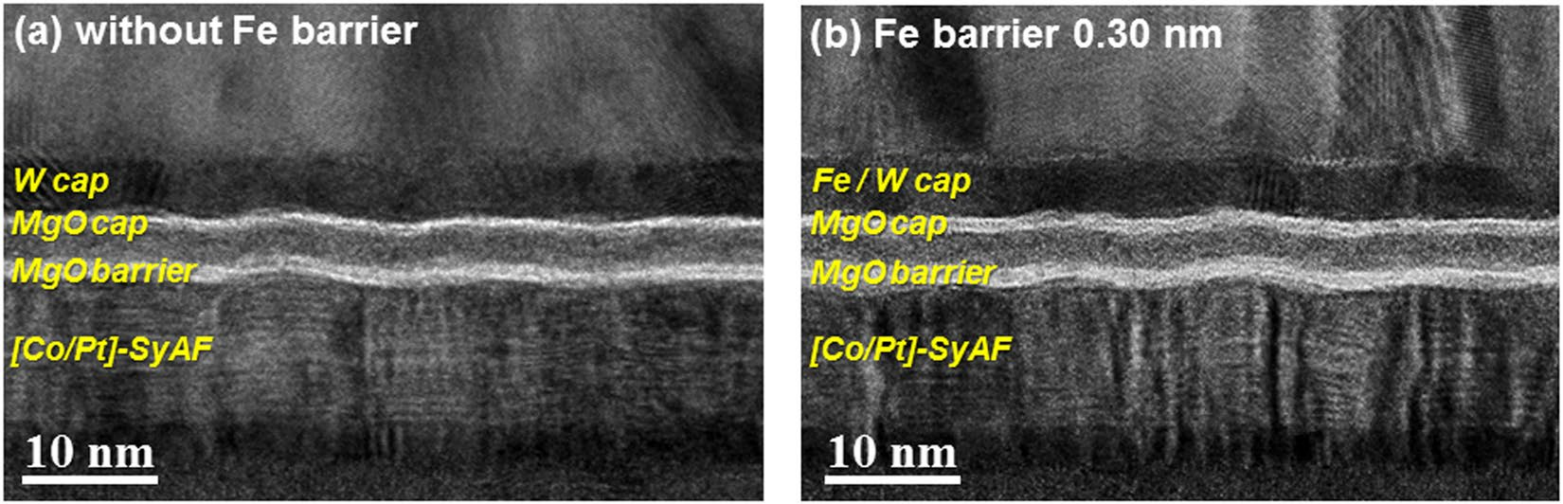
Dependence of the MgO capping and MgO tunneling-barrier thickness distribution on the presence of nanoscale Fe diffusion barrier. Low magnification x-TEM images of p-MTJ spin-valves (**a**) without and (**b**) with 0.3-nm-thick Fe diffusion barrier.

**Figure 5:**
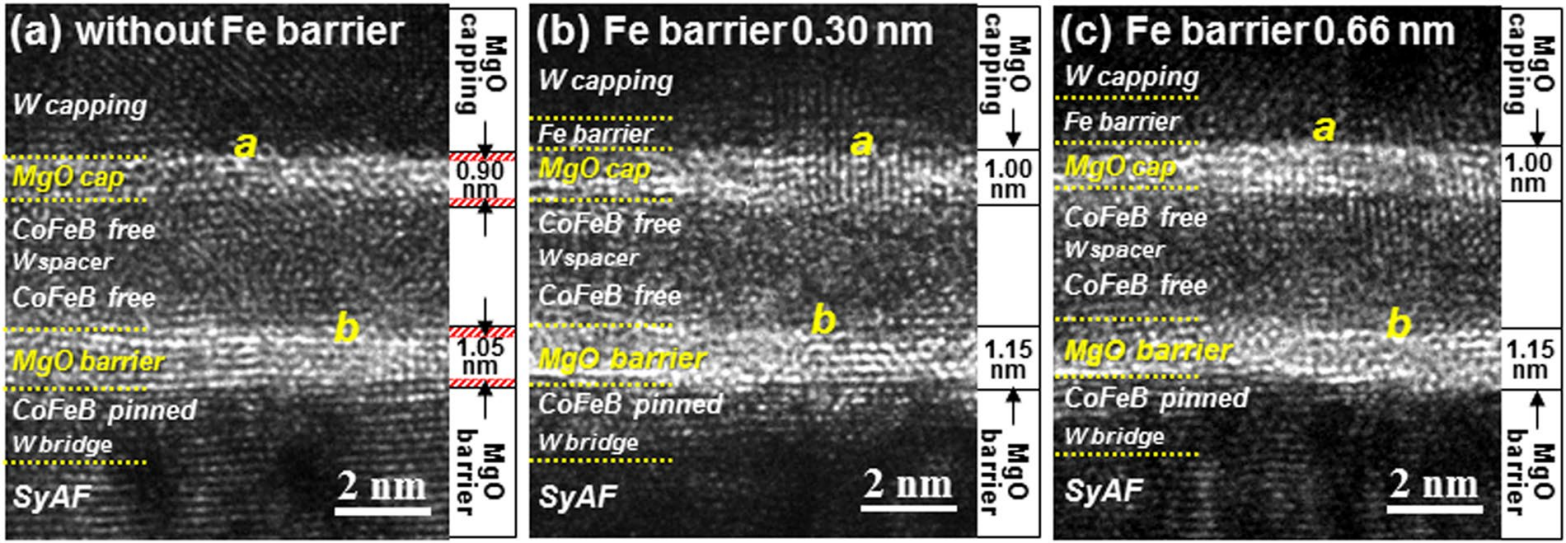
Dependence of f.c.c. crystallinity of the MgO tunneling barrier and MgO capping layer on thickness of Fe diffusion barrier for double MgO-based p-MTJ spin-valves with top Co_2_Fe_6_B_2_ free layer. (**a**) p-MTJ spin-valve without Fe diffusion barrier, (**b**) p-MTJ spin-valve with 0.30-nm-thick Fe diffusion barrier, and (**c**) p-MTJ spin-valve with 0.66-nm-thick Fe diffusion barrier. Cross-sectional high-resolution TEM images were observed at 200 keV in acceleration voltage.

The dependence of the f.c.c. crystallinities of the MgO tunneling barrier and the MgO capping layer in D-p-MTJs on Fe barrier thickness was investigated by x-HR-TEM, (Figs 4 and 5). The double MgO based p-MTJ spin-valve was a vertically stacked structure: a MgO tunneling barrier (1.15 nm)/Fe (0.44 nm)/lower Co_2_Fe_6_B_2_ free layer (1.0 nm)/W spacer layer (0.40 nm)/upper Co_2_Fe_6_B_2_ layer (1.0 nm)/MgO capping layer (1.0 nm)/Fe barrier layer (0–0.66 nm)/W capping layer (4.0 nm). Without the nanoscale diffusion barrier layer, the MgO capping layer looked thinner than the MgO tunneling barrier, (Fig. 4a). It was about 0.90-nm thick and slightly f.c.c. crystallized [**a** in Fig. 5a]; about 0.10 nm was consumed by W diffusion during the *ex-situ* annealing at 400 °C. The MgO tunneling barrier was well f.c.c. crystallized; its thickness was 1.05 nm, and about 0.10 nm had been consumed by W diffusion during the *ex-situ* annealing. On the other hand, for the 0.30-nm-thick Fe barrier layer, the MgO capping layer looked only slightly thinner than the MgO tunneling barrier (Fig. 4b). In particular, a comparison of Fig. 4a with Fig. 4b indicates that the insertion of the diffusion barrier increases the thicknesses of both the capping layer and tunneling barrier. The MgO capping layer was found to be considerably f.c.c. crystallized [**a** in Fig. 5b], and its thickness was 1.00 nm; it was not consumed during the *ex-situ* annealing at 400 °C. The tunneling barrier was also well f.c.c. crystallized and its thickness was 1.15 nm; it was not consumed either. A comparison of Fig. 4a with Fig. 4b indicates that the MgO capping layer and tunneling barrier with the 0.30-nm-thick Fe diffusion barrier were about 0.10-nm thicker than those without the Fe diffusion barrier. Moreover, their f.c.c. crystallinities were slightly better than those without the Fe diffusion barrier. The i-PMA magnetic moment of the double-free-layers (**M**_**a**_) was 217 μemu in the sample with the 0.30-nm-thick Fe diffusion barrier, which is 52 μemu higher than that of the sample without diffusion barrier (165 μemu), as shown in Fig. 1c. As a result, the TMR ratio of the sample with the 0.30-nm-thick barrier (154%) was 23% higher than that of the sample without the barrier (131%) because of the improvement effects of **M**_**a**_ and the f.c.c. crstallinity of the MgO layers (Fig. 1b). For the 0.66-nm-thick Fe diffusion barrier, the MgO capping layer was well f.c.c. crystallized [**a** in Fig. 5c], and its thickness was about 1 nm; it was not consumed during *ex-situ* annealing at 400 °C. In addition, the MgO tunneling barrier was well f.c.c. crystallized, and its thickness was 1.15 nm; it was not consumed either. A comparison of Fig. 5b with Fig. 5c indicates that the thicknesses of the MgO capping layer and the MgO tunneling barrier in the sample with the 0.66-nm-thick Fe barrier were not different from those of the sample with the 0.30-nm-thick barrier. The f.c.c. crystallinity of the MgO capping layer in the case of the 0.66-nm-thick barrier was slightly better than that in the case of the 0.30-nm-thick Fe diffusion-barrier, while f.c.c. crystallinity of the MgO tunneling barrier were not so different for the two cases. Although the f.c.c. crystallinity of the MgO capping layer was slightly better in the case of the 0.66-nm-thick barrier, the i-PMA magnetic moment of the double-free-layers (**M**_**a**_: 193 μemu) was 24 μemu less than in the case of the 0.30-nm-thick Fe barrier (217 μemu), while the IMA magnetic moment of the ferro-coupled free layers (**M**_**IMA**_: 159 μemu) was about 130 μemu higher than in the case with the 0.30-nm-thick barrier (Fig. 1c). As a result, the TMR ratio of the sample with the 0.66-nm-thick barrier (134%) was about 20% lower than that of the sample with the 0.30-nm-thick barrier layer (154%). Note that the Fe concentration increase in the free layer due to diffusion from the Fe layer tended to transform the i-PMA characteristics of the free layer into IMA characteristics so that the TMR ratio rapidly decreased^27^.

### Atomic compositional depth profile

**Figure 6:**
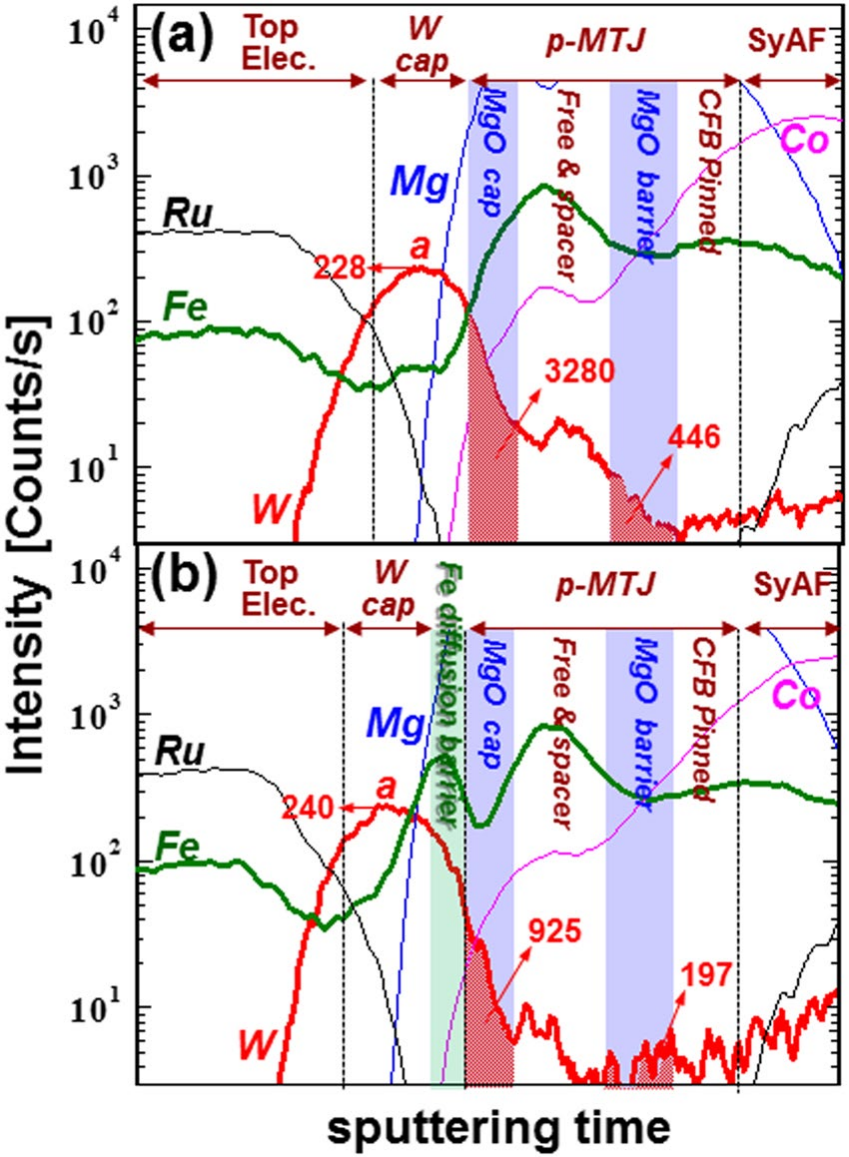
Dependence of W atom compositional depth profile on thickness of Fe diffusion barrier for double MgO-based p-MTJ spin-valves with top Co_2_Fe_6_B_2_ free layer. (**a**) p-MTJ spin-valve without nanoscale-thickness Fe diffusion barrier, (**b**) p-MTJ spin-valve with 0.30-nm-thick Fe diffusion barrier. Free & spacer means the vertically stacked upper Co_2_Fe_6_B_2_ free layer (1.0 nm)/W spacer (0.40 nm)/lower Co_2_Fe_6_B_2_ free layer (1.0 nm)/Fe layer (0.44 nm).

To reveal the reason the 0.30-nm-thick Fe diffusion barrier improved the f.c.c. crystallinity of both the MgO tunneling barrier and capping layer, we investigated its effect on W diffusion by observing atomic compositional depth profiles acquired through HR-SIMS, as shown in Fig. 6. For the spin-valve without the diffusion barrier, the peak relative SIMS count (**a** in Fig. 6a) of W atoms in the 4.00-nm-thick W capping layer was 228, and the areas (relative SIMS count·sputtering time) of the MgO capping and tunneling layers were 3280 and 446, respectively. Note that the peak relative SIMS count and area (relative SIMS count·sputtering time) mean the relative W atom concentration in a layer of nanoscale thickness; i.e., a higher peak relative SIMS count and larger area lead to a higher W atom concentration in the layer. On the other hand, for the spin-valve with the Fe barrier layer, the relative peak count (**a** in Fig. 6b) of W atoms in the capping layer was 240, and the areas of the MgO capping and tunneling layer were 925 and 197 (Fig. 6b). A comparision of the depth profiles in Fig. 6a,b indicates that the relative concentration of W atoms in the W capping layer was slightly higher for the spin-valve with the 0.30-nm-thick Fe diffusion barrier, while the concentration of W atoms in the MgO capping and tunneling layer of the spin-valve with Fe barrier was much lower than that without the barrier. These results mean that inserting a 0.30-nm-thick Fe diffusion barrier between the W capping layer (4.00 nm) and MgO capping layer (1.0 nm) greatly surpresses W diffusion from the W capping layer during *ex-situ* annealing at 400 °C and would thus improve the f.c.c. crystallinity of both the MgO tunneling barrier and capping layer.

**Figure 7:**
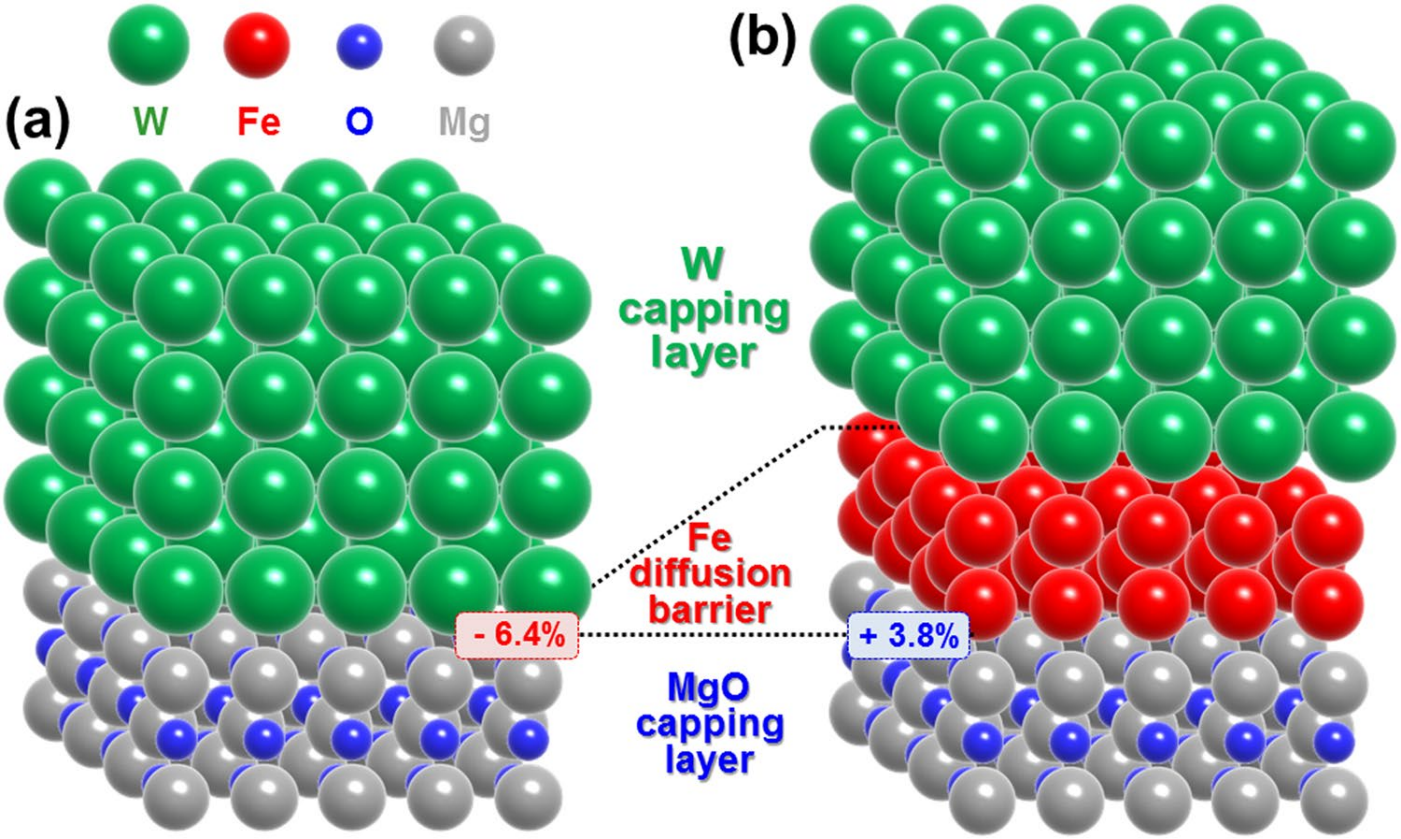
Two types of atomic structure. Atomic structures of (**a**) the interface between the W capping layer and the MgO capping layer and (**b**) the interface between the W capping layer, nanoscale Fe diffusion barrier, and MgO capping layer of double MgO-based p-MTJ spin-valves with top Co_2_Fe_6_B_2_ free layer. Here, (–) corresponds to compressive strain while ( + ) corresponds to tensile strain.

To see how a 0.30-nm-thick Fe diffusion barrier can prevent W from diffusing into the MgO capping and tunneling barrier during *ex-situ* annealing at 400 °C, we calculated the interface strained enhanced diffusivity of W atoms into the MgO capping layer, as shown in Fig. 7. Without a diffusion barrier, the strain at the interface between the W capping layer (4.0 nm) and the MgO capping layer (1.0 nm) was −6.4%, since the lattice constants of the W capping layer and MgO capping layer (tilted 45°) were 316.5 and 297.5 pm, respectively (Fig. 7a)^29^. Note that the atomic diffusivity is exponentially enhanced by the interface strain during *ex-situ* annealing^30^. On the other hand, the interface strain between the Fe diffusion barrier and the MgO capping layer was +3.8%, since the lattice constants of the Fe diffusion barrier and MgO capping layer were 286.6 and 297.9 pm, which is 60% lower interface strain compared with the case without the diffusion barrier (Fig. 7b). Thus, the diffusivity with a 0.3-nm-thick Fe diffusion barrier would likely be much smaller than it would be without such a barrier. As a result, by inserting the diffusion barrier, fewer W atoms would diffuse into the MgO capping and tunneling layers because of the lower W atom diffusivity and longer diffusion distance. In addition, if a lot of W atoms diffuse into the MgO tunneling barrier and capping layer during an *ex-situ* annealing at 400 °C, it would lead to worse f.c.c. crystallinity in both layers because the atomic radius of W (193 pm) is larger than that of magnesium (Mg) (145 nm) and oxygen (O) (48 nm). Therefore, a nanoscale-thickness Fe diffusion barrier could improve the f.c.c. crystallinity of both the MgO tunneling barrier and capping layer since it could reduce the amount of W atoms diffusing into them during *ex-situ* annealing at 400 °C.

### Current-Voltage characteristics of 60-μm p- MTJ spin-valve cells

**Figure 8:**
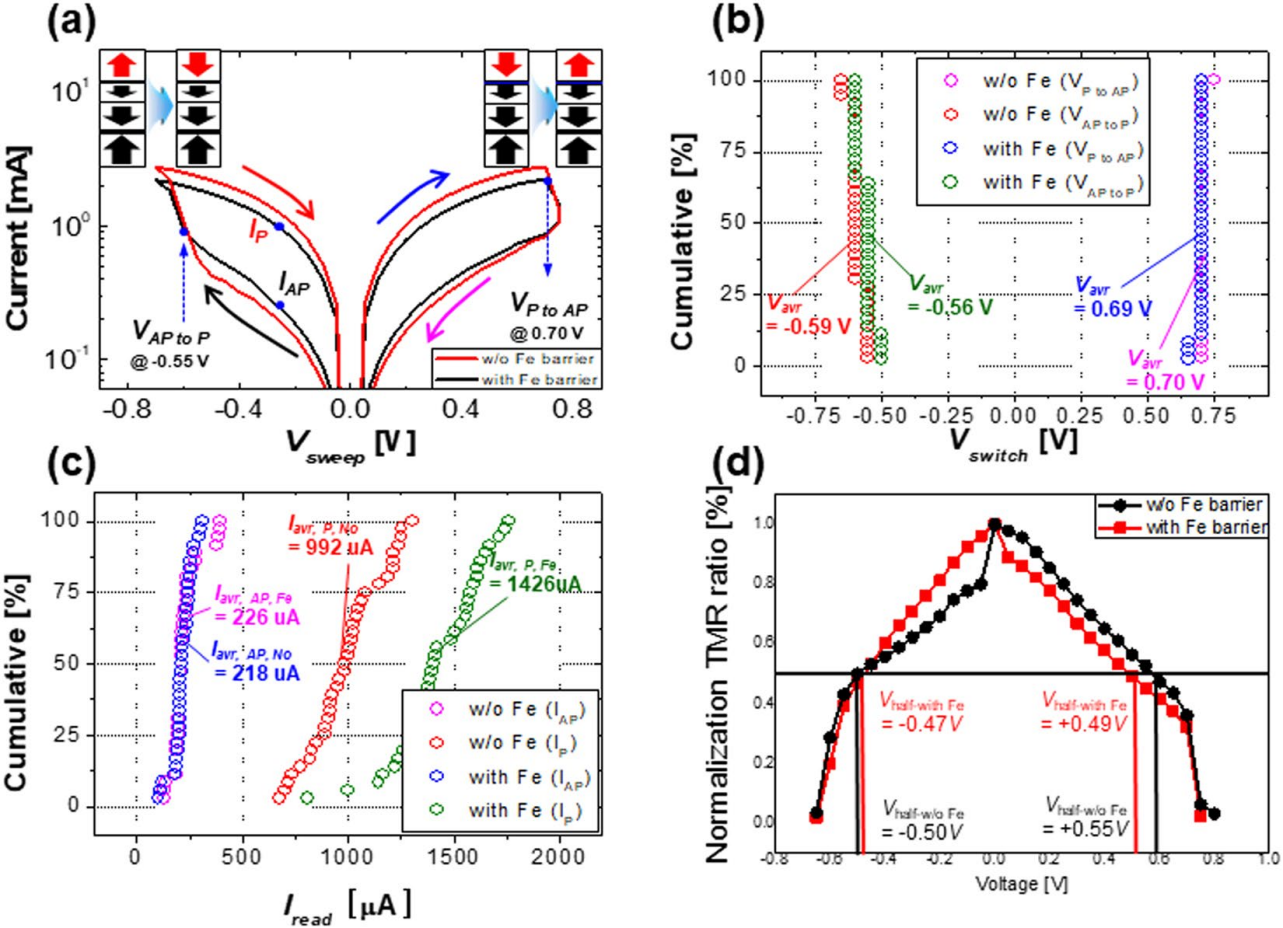
Dependence of nonvolatile memory characteristics of 60-μm p-MTJ spin-valve cells on the presence of nanoscale Fe diffusion barrier. (**a**) I-V curves, (**b**) *V*_*AP to P*_ and *V*_*P to AP*_ distribution, (**c**) *I*_*AP*_ and *I*_*P*_ distribution, and (**d**) Normalized TMR ratio vs. *V*.

To confirm that a nanoscale-thick Fe diffusion barrier improves the f.c.c. crystallinity of both the MgO tunneling barrier and capping layer so as to enhance the nonvolatile memory characteristics of p-STT MRAM cells, we fabricated 60-μm-p-MTJ spin-valve cells with 34-nm-diameter bottom contact electrodes (see also Supplement 5). First, we measured current-versus-voltage (*I-V*) curves for p-MTJ spin-valve cells without and with a 0.3-nm-thick Fe diffusion barrier, where the number of p-MTJ spin-valve cells were 40 for each group. Then, we estimated *V*_*AP* to *P*_ (switching voltage from the anti-parallel to parallel state between the Co_2_Fe_6_B_2_ free and pinned layer), *V*_*P* to *AP*_ (switching voltage from the parallel to anti-parallel state between the Co_2_Fe_6_B_2_ free and pinned layer), the current in the low-resistance state (*I*_*P*_, the reading current in the parallel state between the Co_2_Fe_6_B_2_ free and pinned layer), high-resistance state (*I*_*AP*_, the reading current in the anti-parallel state between the Co_2_Fe_6_B_2_ free and pinned layer) at a reading voltage of −0.25 V, and plotted normalized TMR versus *V* curves from the *I-V* curves, as shown in Fig. 8. Average *V*_*AP* to *P*_ and *V*_*P* to *AP*_ for p-MTJ spin-valve cells without 0.3-nm-thick Fe diffusion barrier were −0.59 V and +0.70 V, while they were −0.56 V and +0.69 V for the cells with the barrier, indicating that the barrier did not influence either *V*_*AP* to *P*_ or *V*_*P* to *AP*_ (Fig. 8b). Otherwise, average *I*_*AP*_ and *I*_*P*_ for cells without Fe diffusion barrier were 226 μA and 992 μA while average *I*_*AP*_ and *I*_*P*_ for cells with Fe diffusion barrier were 218 μA and 1426 μA, implying that the insertion of 0.3-nm-thick barrier did not affect *I*_*AP*_ but intensively enhanced *I*_*P*_, as shown Fig. 8c. Figure 8d represents the normalized TMR versus *V* curves in which the voltages where the TMR ratio obtains half of the maximum value (*V*_*half-with Fe*_ and *V*_*half-w/o Fe*_) were estimated to be −0.50 *V* and 0.55 *V* for cells without nanoscale Fe diffusion barrier, while the voltage of cells with 0.3-nm-thick Fe diffusion barrier were −0.47 *V* and 0.49 *V*. This implies that the insertion of the 0.3-nm-thick Fe diffusion barrier enhanced the tunneling coherence ability of the p-MTJ spin-valve cells. Note that the amount of symmetric behavior of normailized TMR ratio indicate the achievement degree of the coherence tunneling of the p-MTJ spin-valve^31^.These nonvolatile memory characteristics of 60-μm p-MTJ spin-valve cells (Fig. 8) were well correlated with the f.c.c. crystallinity of both the MgO tunneling barrier and capping layer (Figs 4 and 5). Since the insertion of the diffusion barrier improves the f.c.c. crystallinity of both the MgO tunneling barrier and capping layer, it greatly increases the reading current (*I*_*P*_) in the parallel state, but did not change the reading current (*I*_*AP*_) in the anti-parallel state. Note that the magnitude of *I*_*P*_ is mainly determined by the i-PMA magnetic moment of the Co_2_Fe_6_B_2_ free layer because a higher i-PMA magnetic moment leads to a higher filtered perpendicular spin-torque current through the MgO tunneling barrier with *Δ*_1_ coherent tunneling. However, the magnitude of *I*_*AP*_ is not influenced much by the i-PMA magnetic moment of the Co_2_Fe_6_B_2_ free layer because most of the perpendicular spin-electrons tunneling in the anti-parallel state between the Co_2_Fe_6_B_2_ free and pinned layer are reflected at the interface between the Co_2_Fe_6_B_2_ free layer and the MgO barrier. Thus, the insertion of 0.3-nm-thick Fe diffusion barrier remarkably increases the TMR ratio of p-MTJ spin-valve cells improving the f.c.c. crystallinity of both the MgO tunneling barrier and capping layer of p-MTJ spin-valves and therby increasing *I*_*P*_.

## Discussion

For tera-bit intergration of p-STT MRAM cells, D-p-MTJ may be a way to provide a high TMR ratio and high thermal stability. However, the TMR ratio of such spin-valves with a top free Co_2_Fe_6_B_2_ layer decreases rapidly at 400 °C (BEOL)^32^. A novel solution to this problem is the insertion of a nanoscale Fe diffusion barrier (only two atomic Fe layers) between the W capping layer (i.e., 4.00-nm thick) and MgO capping layer (1.00-nm thick); it can dramatically reduce the amount of W atoms diffusing into the MgO tunneling barrier and capping layers and remarkably improve the crystallinity of these layers; thereby, it can greatly enhance the TMR ratio at BEOL temperature of 400 °C. However, the TMR ratio is extremely sensitive to the thickness of the Fe diffusion barrier; i.e, it peaked (154%) at a thickness of only two atomic Fe layers (0.3 nm). In particular, it tends to decrease when the Fe diffusion barrier layer exceeds this thickness, since the ferro-magnetic Co_2_Fe_6_B_2_ tends to transform the i-PMA characteristic into an IMA characteristic. In addition, inserting a Fe diffusion barrier would solve the problem of i-PMA characteristic degradation of the Co_2_Fe_6_B_2_ pinned layer of these spin-valves *ex-situ* annealed at 400 °C (BEOL); inserting one between the Co_2_Fe_6_B_2_ pinned layer and W bridging layer in Fig. 1a would reduce W diffusion from the W bridging layer and Pt diffusion from the [Co/Pt]_n_ SyAF layer. Thus, the design of double MgO-based p-MTJ spin-valves utilizing a nanoscale-thickness Fe diffusion barrier would be a key to achieving a high TMR ratio and high thermal stability simultaneously at the BEOL of 400 °C.

## Methods

The double MgO-based p-MTJ spin-valves used in our experiments were fabricated on 12-inch-diameter Si/SiO_2_ wafers by using a 12-inch-wafer multi-chamber sputtering system kept at 1 × 10^−8^ torr without breaking the high vacuum, as shown in Fig. 1a. They were vertically fabricated on a [Co/Pt]_n_-based synthetic-anti-ferro-magnetic (SyAF) layer, and hence, they are called double MgO-based p-MTJ spin-valves with a top free Co_2_Fe_6_B_2_ layer. In addition, W was used as a nanoscale-thickness bridge layer (~0.24 nm), spacer layer (~0.40 nm), and capping (~4.00 nm) layer. A nanoscale-thickness Fe diffusion barrier with a b.c.c. crsytallized structure was inserted between the W and MgO capping layer (~1.00 nm) and its thickness was varied over the samples between 0 and 0.66 nm. All of the spin-valves were subject to *ex-situ* annealing at 400 °C for 30 min under a perpendicular magnetic field of 3 Tesla.

## Electronic supplementary material

Supplementary Information
