# Supplementary material for: Highly Enhanced TMR Ratio and Δ for Double MgO-based p-MTJ Spin-Valves with Top Co2Fe6B2 Free Layer by Nanoscale-thick Iron Diffusion-barrier
Source: Sci Rep. 2017 Sep 19;7:11907. doi: 10.1038/s41598-017-10967-x (PMC5605541; doi:10.1038/s41598-017-10967-x)
Supplement: Supplementary file 1 — Supplementary Information [file 41598_2017_10967_MOESM1_ESM.doc]

Supplementary Information for

**Highly Enhanced TMR Ratio and Δ for Double MgO-based p-MTJ Spin-Valves with Top Co_2_Fe_6_B_2_ Free Layer by Nanoscale-thick Iron Diffusion-barrier**

**Seung-Eun Lee^a^, Jong-Ung Baek^b^, and Jea-Gun Park^*a^**

a) MRAM Center, Department of Electronics and Computer Engineering, Hanyang University, Seoul, 04763, Republic of Korea
b) MRAM Center Department of Nanoscale Semiconductor Engineering, Hanyang University, Seoul, 04763, Republic of Korea

*parkjgl@hanyang.ac.kr

**Supplement 1. Dependency of the TMR ratio on the spacer material and its thickness for double MgO-based p-MTJ spin-valves with a top Co_2_Fe_6_B_2_ free layer. (a) Ta spacer and (b) W spacer.**

***Supplement 1***

**
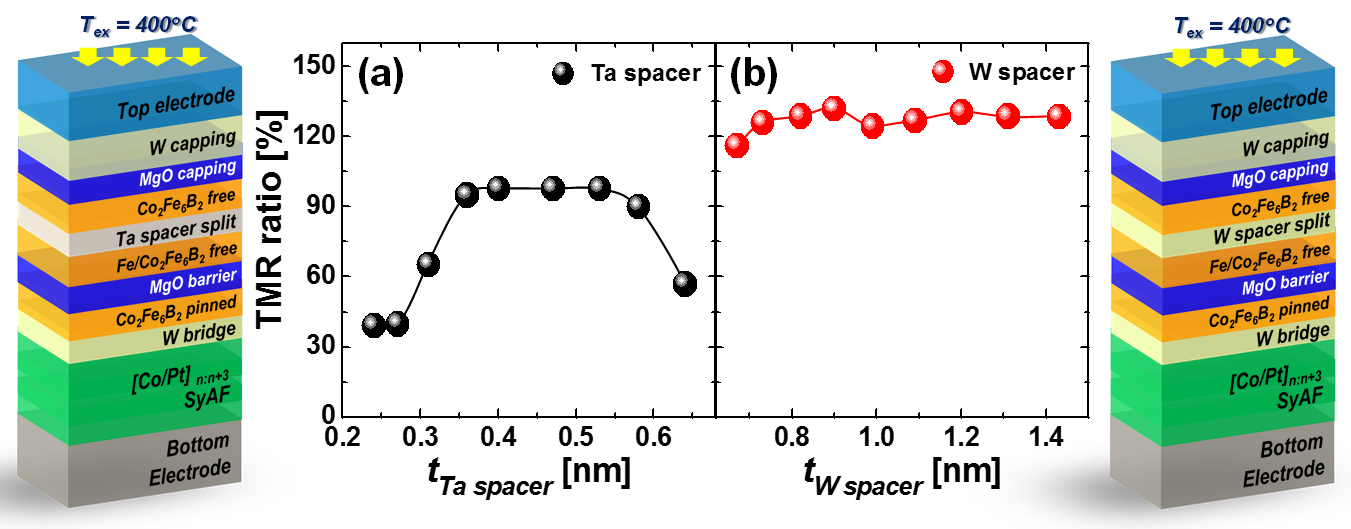
**

**Supplement 2. XRD spectra of Fe diffusion barrier for various thickness; Sub/MgO(1.0)/Fe(x) thickness in nanometers**

***Supplement 2***

**
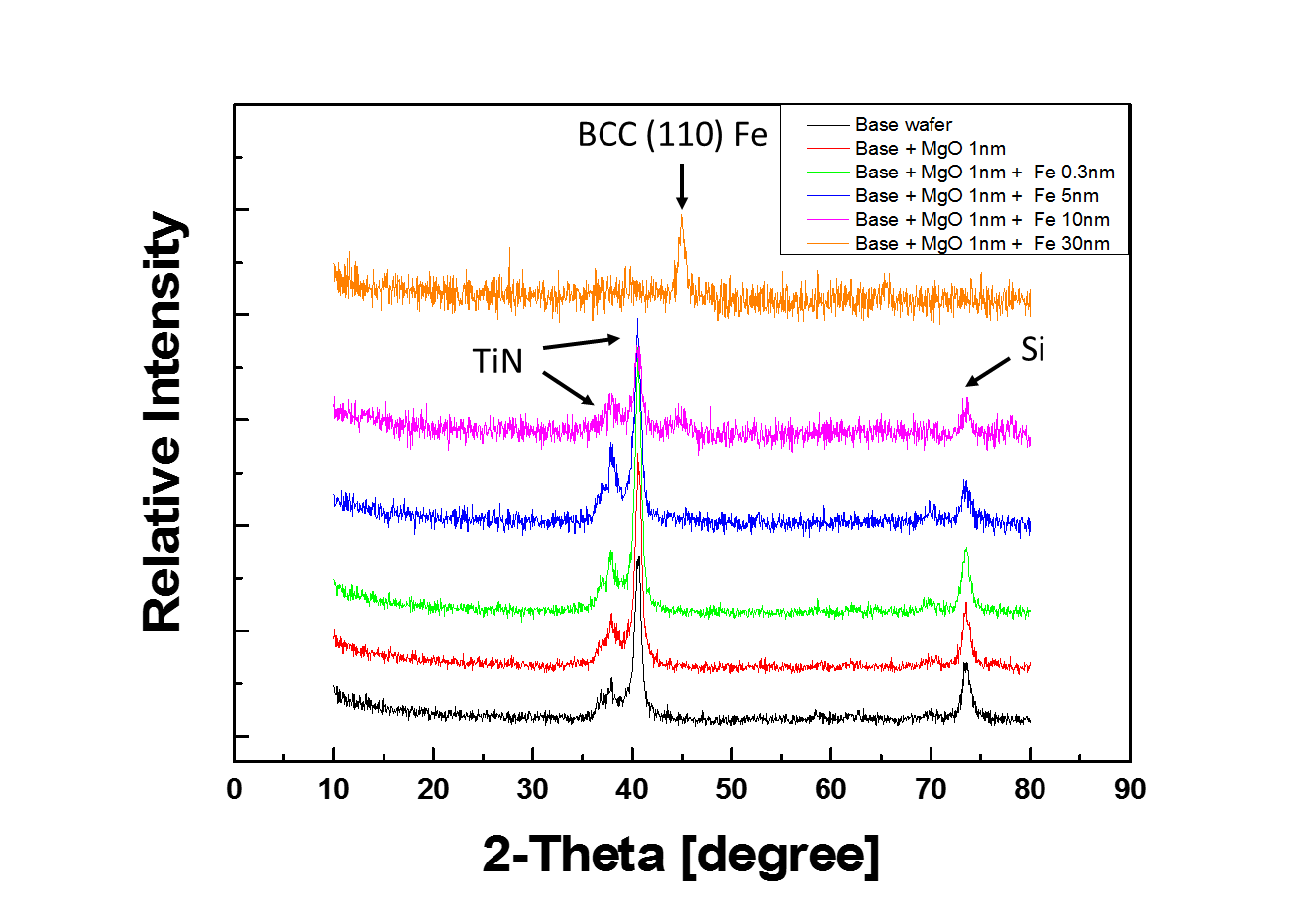
**

**Supplement 3. (a) p-MTJ spin-valve structure, (b) the TMR ratio measured by CIPT and (c) the TMR ratio measured by p-MTJ spin-valve cell with 250-nm diameter.**

***Supplement 3***

**
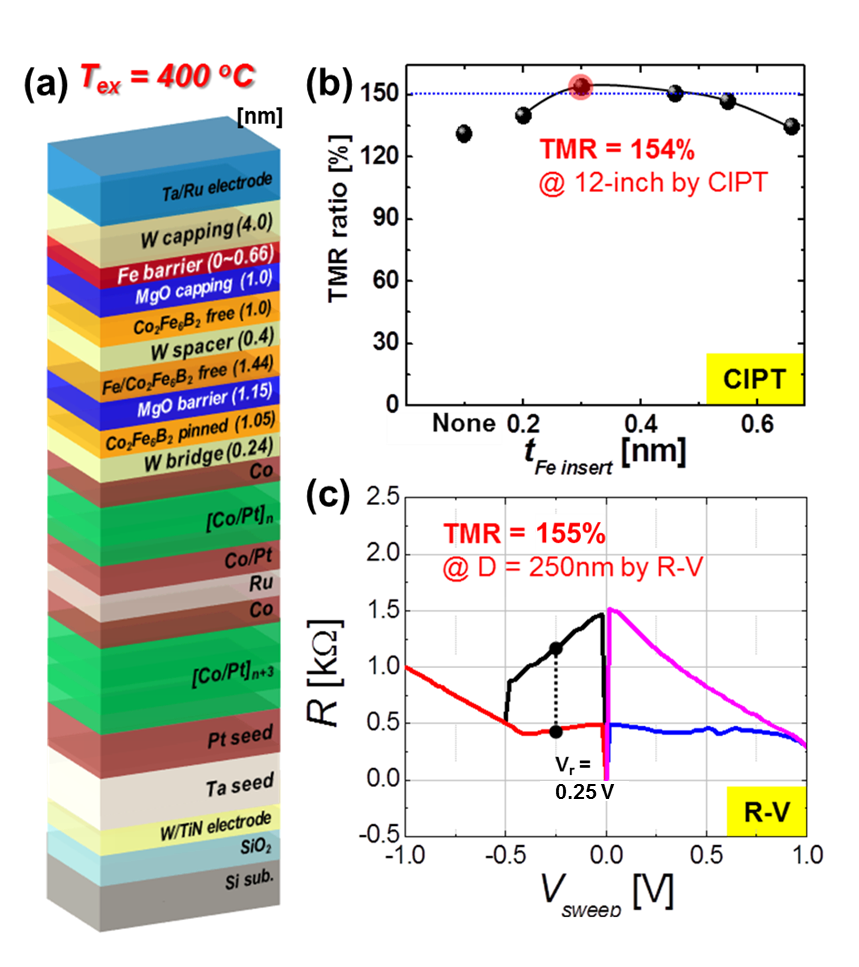
**

**Supplement 4. Experimental values (*t*_eff_, *M*_s_, *H*_k_)for thermal stability of p-MTJ spin-valves (a) with and (b) without Fe diffusion barrier**

***Supplement 4***

**
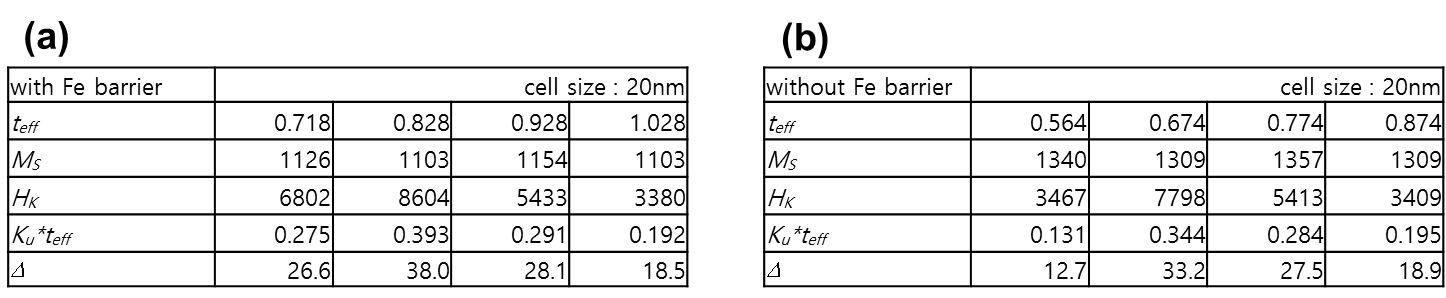
**

**Supplement 5. Schematic drawing of 30-nm-diameter p-MTJ spin-valve cell-array. The p-MTJ spin valve cells were fabricated with a vertical stacking of a TiN bottom electrode/p-MTJ spin valve/Ru top electrode, which was the same vertical structure of p-MTJ spin-valves for the CIPT measurement in Fig. 1(a).**

***Supplement 5***

**
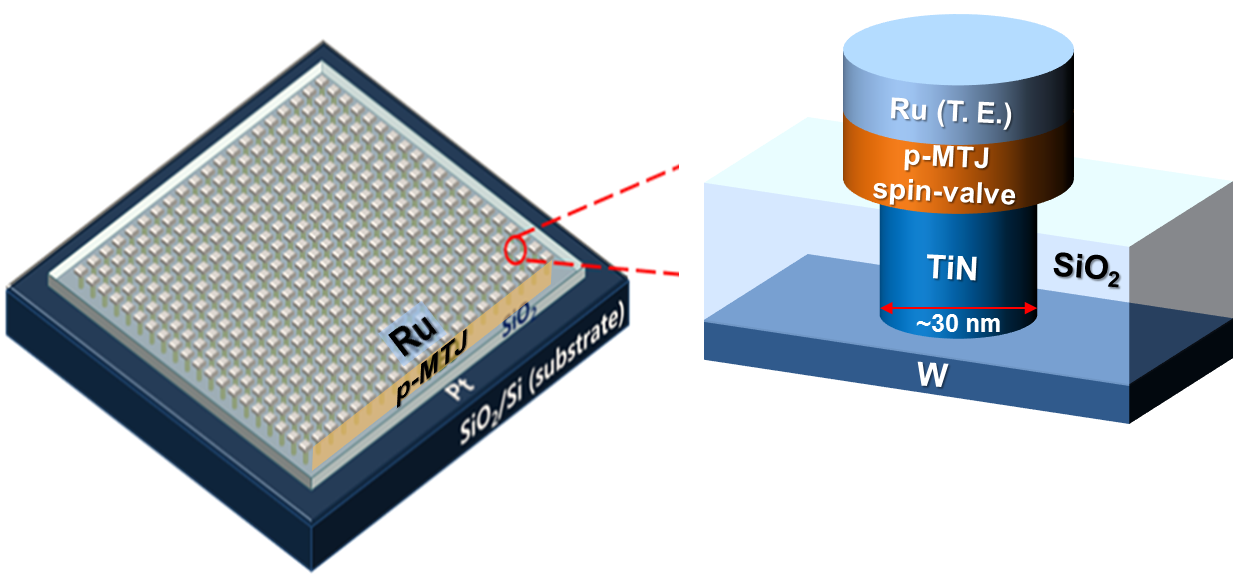
**
